# Genetic determinants of methotrexate treatment efficacy in patients with juvenile idiopathic arthritis

**DOI:** 10.1186/1546-0096-12-S1-P26

**Published:** 2014-09-17

**Authors:** Mojca Zajc Avramovič, Nataša Toplak, Meta Accetto, Maruša Debeljak, Lara Lusa, Vita Dolžan, Tadej Avčin

**Affiliations:** 1 Paediatric Clinic of University Clinical Centre Ljubljana, Slovenia; 2 Institute for Biostatistics and Medical Informatics; 3 Institute for Biochemistry, Medical Faculty, Ljubljana, Slovenia

## Introduction

Single nucleotide polymorphisms (SNPs) are common (1%) variations in DNA sequence, that can be the reason for individual variability in drug efficacy and drug safety.

## Objectives

To investigate the effect of single nucleotide polymorphisms in the genes for methotrexate (MTX) uptake and efflux mechanisms and in the genes of the adenosine pathway on response to therapy in JIA.

## Methods

The data of 116 consecutive patients with JIA treated with MTX at the University Children’s Hospital Ljubljana from June 2011 to May 2014 have been retrospectively reviewed. The disease activity was measured by JADAS 71

score 3 and 6 months after the beginning of treatment with MTX and at the last follow up visit. All adverse events were noted separately for different organ systems. Genotyping of single nucleotide polymorphisms (SNP) in the genes of MTX transporters and in the adenosine pathway was performed using real time PCR methods. The following SNPs were analyzed: ABCB1 3435C>T (rs1045642),ABCC2 24C>T (rs717620), ABCC2 1019A>G (rs2804402), ABCC2 1249G>A (rs2273697), ABCG2 34G>A (rs2231137),ABCG2 421C>A (rs 2231142), SLCO1B1 174Ala>Val (rs4149056), SLCO1B1 388 A>G (rs2306283), SLCO1B1 int13 T>C (rs11045879), SLC19A1 (RFC1) 80G>A (rs1051266), ATIC (347C>G), AMPD (34C>T) and ITPA (94A>C). Kaplan Meier estimater and penalized Cox regression model were used for statistical analysis.

## Results

The study group included 88 (76%) girls and 28 (24%) boys with JIA. 10 (9%) patients had systemic arthritis, 43 (37%) patients had polyarthritis (5 out of these were RF positive), 25 (22%) patients had persistent oligoarthritis, 22 (19%) extended oligoarthritis, 10 (9%) patients had juvenile psoriatic arthritis and 2 (2%) patients suffered from enthesitis related arthritis. Mean follow up time was 80 months. 75 (65%) patients were switched to higher dosage od methotrexate to achieve inactive disease. In total 52 (45%) patients had to be switched to biologic therapy due to treatment inefficacy or severe adverse events. Mean treatment duration until switching to biologic therapy was 17,5 months. Adverse events developed in 70 (60%) patients, 14 (12%) patients had severe adverse events and 10 (9%) patients discontinued MTX treatment because of adverse events. 16 (14%) patients were in remission without therapy at the last follow up visit.

Using Kaplan Meier estimater ABCB1 3435C>T (rs1045642) and ABCC2 1249G>A (rs2273697) were associated with probability of starting biological treatment (P=0,1 and P=0,15). Using a penalized Cox regression model, ABCC2 1249G>A (rs2273697) was confirmed to be found associated to probability of starting biological treatment (HR=1.09 mutated vs wt).

## Conclusion

ABCC2 1249G>A (rs2273697) could be a useful early predictor for MTX treatment inefficacy. SNPs in MTX transporter genes and in the adenosine pathway could be factors to predict treatment outcome, but more studies need to be done.

## Disclosure of interest

None declared.

